# Single Versus Two Plate Osteosynthesis for Parasymphysis Fractures of the Mandible: A Prospective Comparative Study

**DOI:** 10.7759/cureus.35311

**Published:** 2023-02-22

**Authors:** Kamalakannan Padmanaban, Durairaj Duraisamy, Davidson Rajiah, Priyadharshini Raghavan, Senthilkumar Annamalai

**Affiliations:** 1 Department of Oral and Maxillofacial Surgery, Tamil Nadu Government Dental College and Hospital, Chennai, IND; 2 Department of Oral and Maxillofacial Surgery, Adhiparasakthi Dental College and Hospital, Melmaruvathur, IND

**Keywords:** parasymphysis fractures, miniplates, mandibular fracture, lines of osteosynthesis, arch bar

## Abstract

Background

The treatment of mandibular fractures has undergone a revolutionary change after the invention of miniplate osteosynthesis. There aren't many studies in the literature comparing the outcomes of treating mandibular parasymphysis fractures with two miniplates versus one miniplate.

Aim

To evaluate the outcomes of single vs. two plate osteosynthesis in the management of parasymphysis fractures of the mandible.

Materials and methods

Sixteen patients with parasymphysis fracture of the mandible treated with a single high-profile miniplate or with two miniplates were included in the study. Multiple outcome variables such as fracture union, wound infection, dehiscence, iatrogenic dental injury, intraoperative time, plate exposure, and need for plate removal were recorded and analyzed statistically using the Student's t-test.

Results

The occurrence of iatrogenic dental injury (P= 0.021) and the mean operating time showed a statistically significant difference between the groups, whereas variables such as osseous healing, plate exposure, wound dehiscence, and postoperative paresthesia had no significant difference (P > 0.05).

Conclusion

Despite both systems providing enough stability for osseous healing, the use of a single high-profile miniplate demonstrated fewer post-operative problems, such as iatrogenic injury to the dental roots and occurrence of infection/dehiscence and plate exposure, than the use of two miniplates.

## Introduction

Miniplate osteosynthesis has brought about a revolutionary change in the management of mandibular fractures. It is based on the principles of Champy’s ideal lines of osteosynthesis [1], which advocates the placement of a miniplate along the lines of tension at the upper border of the mandible. However, in the symphysis and parasymphysis region of the mandible, it recommends the placement of two miniplates, one on the upper border and one on the lower border, to counteract the high torsional forces in this region [1]. Placement of miniplates in the upper border at the canine-premolar region of the mandible is associated with an increased risk of injury to the inferior alveolar/mental nerve and dental roots, often due to the presence of insufficient space between these structures [2]. In such situations, one might choose to use a single bone plate along the inferior border rather than risk injury to the nerves or teeth when placing the second bone plate [2]. Yet, to neutralize the tensional forces in the upper border of the mandible and to obtain a functionally stable fixation [1], a tension band in the form of an arch bar is always needed. There are only a few studies in the literature comparing the outcomes of using two miniplates and a single high-profile plate with an arch bar for the management of mandibular parasymphysis fractures. Hence, in this study, these two methods of osteosynthesis are comparatively evaluated.

## Materials and methods

This prospective comparative study was presented and approved by the institutional review board of the Tamil Nadu government dental college and hospital, Chennai (Ref. No.: 0430/DE/2010, Date of approval: 10/03/2013). The study was carried out on 16 patients with mandibular fractures reported to the Department of Oral and Maxillofacial Surgery between the period of January 2013 and November 2013.

Inclusion criteria

Healthy, dentate individuals of 15-55 years of age, of both sexes with a simple (linear and non-comminuted) fracture in the parasymphysis region of the mandible and without any signs/symptoms of mental nerve injury (preoperatively) were included in the study.

Exclusion criteria

Patients with severely comminuted/infected fractures, associated bilateral condylar fractures (treated by closed reduction), associated midface fractures that alter the occlusion, and medically compromised patients were excluded from the study.

**Figure 1:**
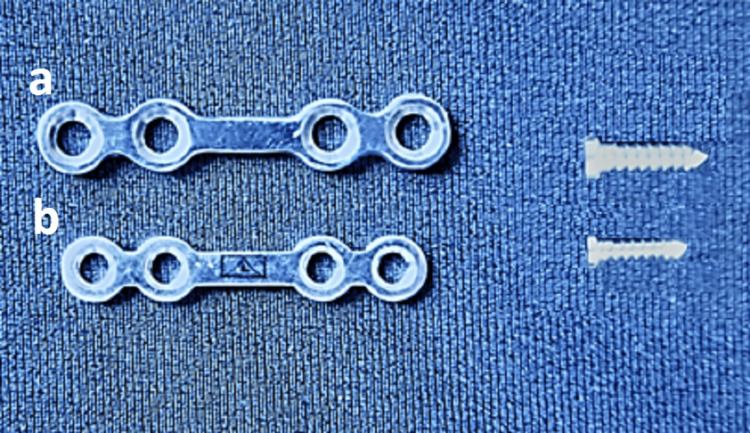
Comparison of the two osteosynthesis materials used in the study: a) 2.5 x 4-hole miniplate and 10-millimeter screw, b) 2 x 4-hole miniplate and 8-millimeter screw

**Figure 2:**
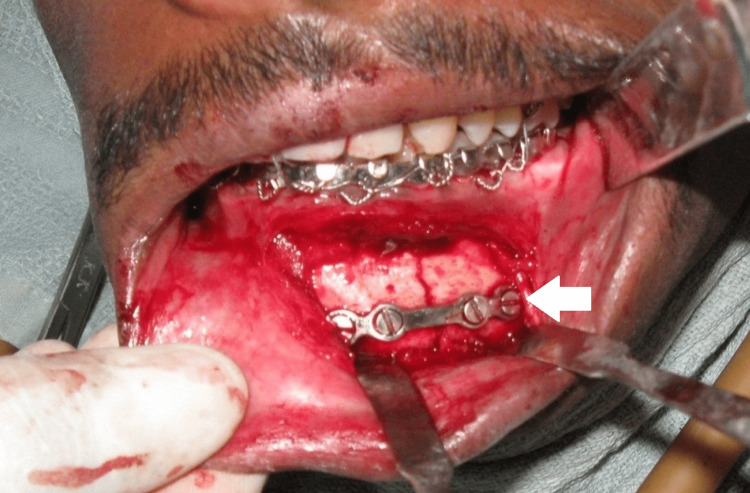
Reduction and fixation of parasymphysis fracture in Group I using a single 2.5 × 4-hole miniplate and 10-millimeter screws

**Figure 3:**
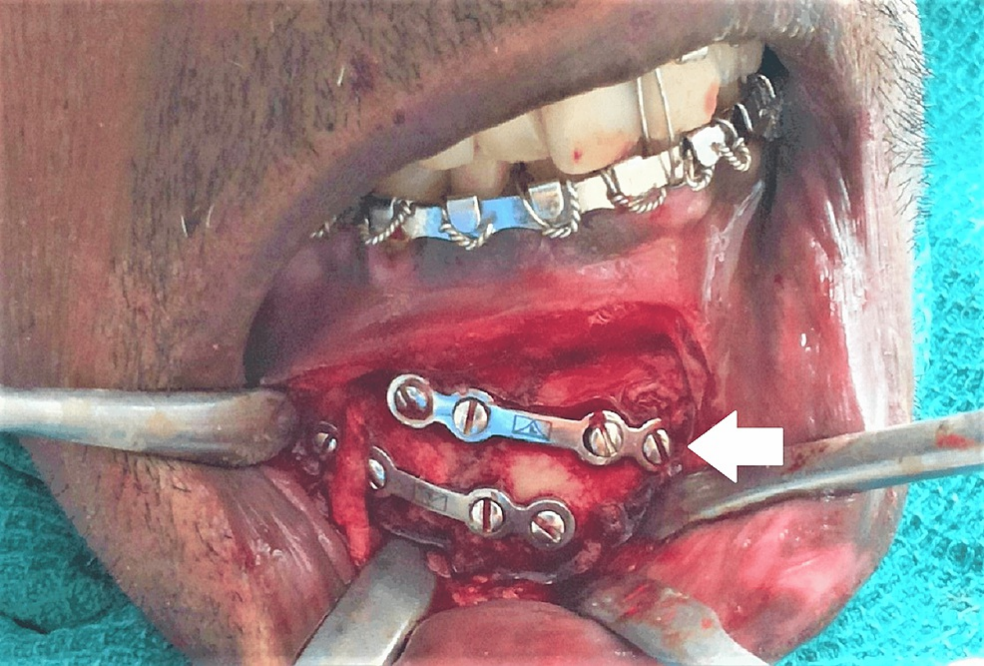
Reduction and fixation of parasymphysis fracture in Group II using two 2 x 4-hole miniplates and 8-millimeter screws

All patients were randomly categorized into two groups (eight in each group). Group I patients are treated with a single 2.5 × 4-hole miniplate and 10-millimeter screws, while Group II patients are treated with two 2 × 4-hole miniplate and 8-millimeter screws (Figures 1-3). All the procedures followed were in accordance with the ethical standards of the responsible committee on human experimentation. Upper and lower arch bars were placed in both groups preoperatively, and maxillo-mandibular fixation was kept in place for one week postoperatively. The lower arch bar was maintained for five weeks postoperatively in Group I patients alone.

Surgical procedure

After obtaining written informed consent, all patients were posted for surgery. The face was painted with a dilute solution of povidone-iodine. 2% lignocaine with 1:200000 adrenaline was used as the local anesthetic agent. Erich arch bars were placed in both the upper and lower arches. The fracture site was exposed through an intraoral mandibular vestibular incision [3] extending on either side of the fracture line. Mentalis muscle was incised, and subperiosteal dissection was carried out to the lower border of the mandible. The fracture site was identified and reduced. Occlusion was established with maxillo-mandibular fixation. Reduced fragments were fixed by a single 2.5 × 4-hole miniplate and four 2.5 × 10-millimeter screws in the lower border in Group I and by two 2 × 4-hole miniplates and eight 2 × 8-millimeter screws in the lower border and at the base of the alveolar ridge (2 millimeters below the root apices) in Group II. Occlusion was rechecked. The operative site was irrigated with betadine and saline and sutured with 3-0 vicryl and 3-0 silk. An extraoral pressure bandage was applied. All patients were kept under antibiotic coverage [beta-lactams (Cefotaxime/cephalexin/amoxicillin) and metronidazole] for one week. They were advised to take a liquid diet for one week and, thereafter, a soft diet for the next four weeks. They were also instructed to use chlorhexidine mouth rinse frequently and to maintain their oral hygiene. Both groups of patients were kept under post-operative maxillo-mandibular fixation for one week. In the event of associated unilateral condyle fractures, the maxillo-mandibular fixation period was extended for three weeks (closed management). Sutures were removed on the seventh post-operative day, and patients were reviewed periodically.

Follow-up and observation

All the patients were followed up at one-week intervals for the first five weeks and once a month thereafter for the next six months. Radiographs were taken on the second postoperative day, at the end of five weeks, and the end of six months. Patients are evaluated for fracture union (clinical and radiographic), wound infection and dehiscence, plate exposure/need for plate removal (as evident by the presence of pain, pus discharge, swelling, or radiographic loosening of screws), iatrogenic dental injury (as evident in the post-operative radiograph), anesthesia or paresthesia due to mental nerve injury (as evaluated using static light touch, two-point discrimination, brush directional discrimination, and pin pressure methods) [4] and intraoperative time. The observations obtained were evaluated statistically using Student’s t-test.

## Results

**Figure 4:**
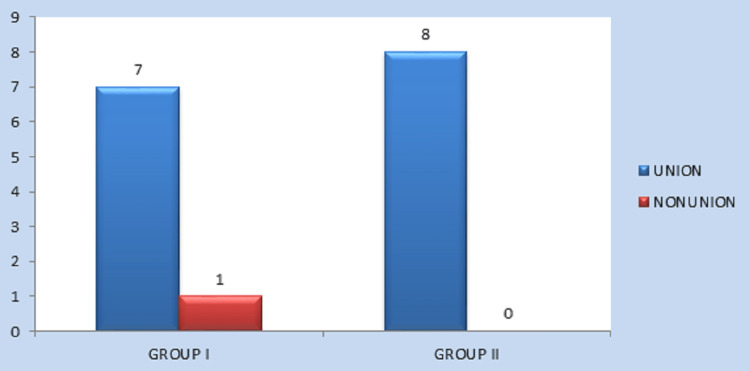
Comparison of fracture union between Group I and Group II at the end of five weeks

The mean age of patients in Group I was 29.38, and in Group II was 24.38, with seven males (87.5%) and one female (12.5%) in each group. Evaluation of fracture union at the end of five weeks showed that seven patients (87.5%) in Group I and all eight patients (100%) in Group II exhibited good fracture union. Only one patient (12.5%) in Group I showed signs of non-union. However, the difference (P = 0.302) was not statistically significant (Table 1) (Figure 4).

**Table 1:** Outcome variables *Mean operating time

Outcome variable	Group I		Group II		P-value
	Number of patients	Percentage	Number of patients	Percentage	
Fracture non-union	1	12.5	0	0	0.302
Wound infection/dehiscence	0	0	2	25	0.131
Plate exposure	0	0	1	12.5	0.302
Iatrogenic dental injury	0	0	4	50	0.021
Paraesthesia	2	25	3	37.5	0.590
Time		*72 minutes		*84 minutes	0.00

**Figure 5:**
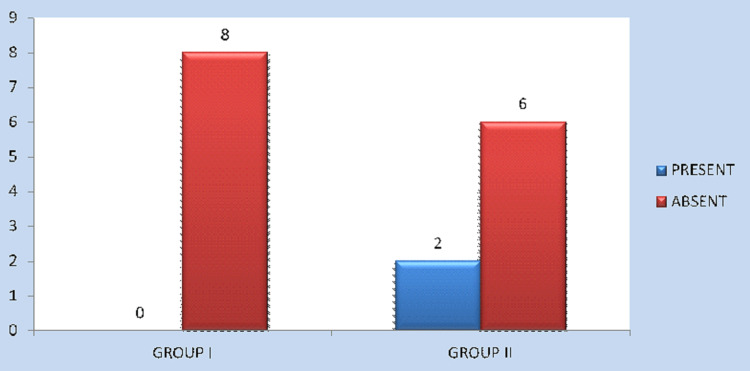
Occurrence of wound infection/dehiscence in Group I and Group II

**Figure 6:**
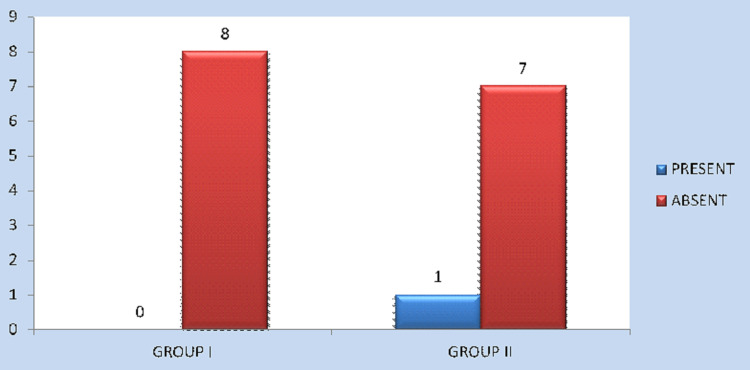
Occurrence of plate exposure in Group I and Group II

Wound dehiscence occurred in none of the patients (0%) in Group I and two patients (25%) in Group II with P = 0.131, which is not statistically significant. Similarly, none of the patients in Group I and one patient in Group II (12.5%) needed plate removal due to the loosening of screws and subsequent infection, and this was also not statistically significant (Figures 5, 6).

**Figure 7:**
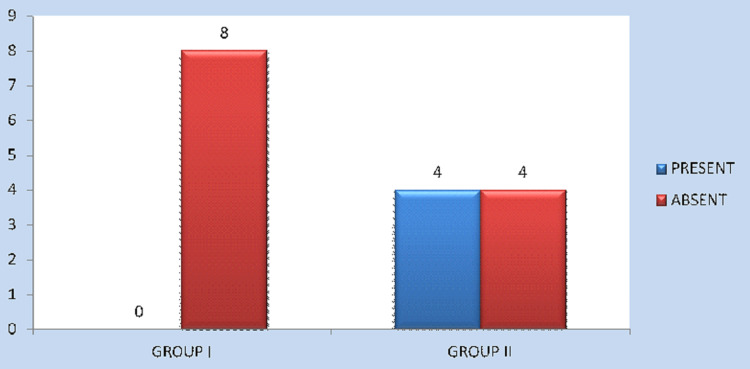
Incidence of iatrogenic dental injury among Group I and Group II patients

**Figure 8:**
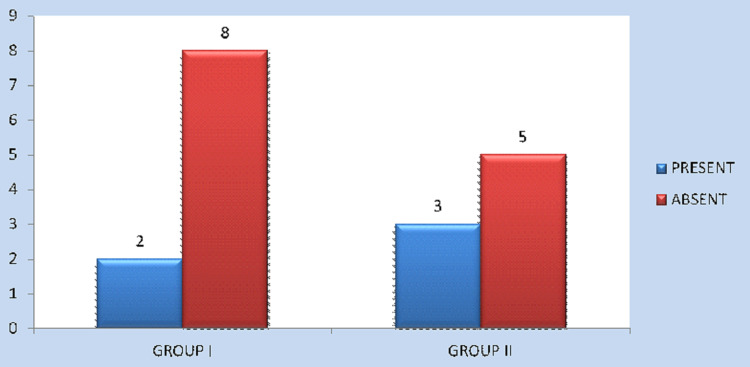
Occurrence of post-operative paraesthesia among Group I and Group II patients

**Figure 9:**
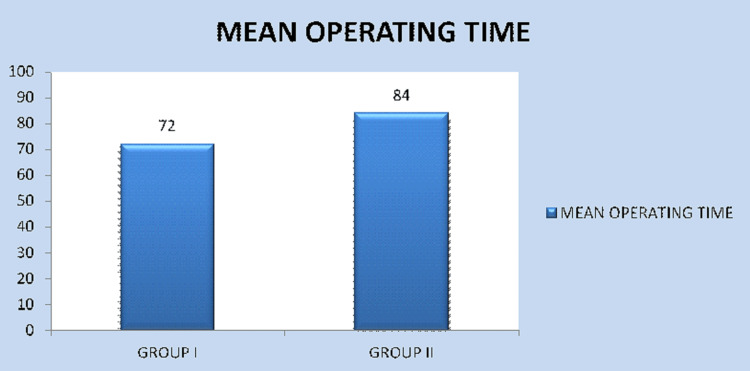
Comparison of mean operating time (minutes) between Group I and Group II

As far as the iatrogenic dental injury is concerned, four cases (50%) in Group II exhibited iatrogenic dental injury, while it occurred in none of the cases in Group I (0%); the results (P = 0.021) were statistically significant (Figure 7). Post-operative paraesthesia occurred in two patients (25%) in Group I and three patients (37.5%) in Group II. P = 0.590 and was not statistically significant (Figure 8). The mean intraoperative time was 72 minutes in Group I and 84 minutes in Group II. The p-value was 0.00 and was highly significant (Figure 9).

## Discussion

Fractures of the mandible occur more frequently than fractures of the other facial bones due to its prominent peripheral position around the lower third of the facial skeleton [5-7]. Mandibular fracture management focuses on the early regain of pre-injury form and function with as minimal morbidity as possible [8]. In 1973, Michelet et al. [1] pioneered the use of miniplates for mid-face fractures, followed by Champy et al. for the mandible [1]. Studies on the biomechanical characteristics of horizontal ramus revealed that the masticatory pressures generated within the mandible elicit elongation stress along the alveolar border and compression along the lower border [9]. In light of this, they discussed the idea of mandibular fixation employing monocortical juxta-alveolar and sub-apical osteosynthesis without compression and intermaxillary fixation. Furthermore, the placement of two miniplates (one subapically and one at the lower border) in the mandibular anterior region was advocated to counteract the torsional forces, which are high in this region due to muscle attachments on the lingual aspect of the mandible.

However, various studies on the course of the inferior alveolar nerve within the mandible and lateral cortical bone thickness in the canine/premolar region indicate that the placement of miniplates in the subapical portion of this region is always associated with the risk of injury to root apices or to inferior alveolar/mental nerves [10]. Although Champy [1] did not use arch bars for intraoperative intermaxillary fixation, most surgeons use arch bars either for intra- or post-operative intermaxillary fixation to achieve more stable occlusion. The lower arch bar placed for this purpose itself acts as a tension band, and the need for a subapical plate can be eliminated [8]. Furthermore, placing a plate on the upper border increases the risk of wound infection, dehiscence, and plate exposure. Hence, the management of parasymphysis fracture with a miniplate at the inferior border and an arch bar at the alveolar segment seems to be a more logical approach.

Demographic information

The predominance of mandibular fractures in men is a relatively consistent finding in most studies, and it appears to be the same in ours, with 14 of the total 16 patients being male (87.5%) and the remaining two being female (12.5%). In a retrospective analysis of 162 patients, it was found that the fractures occurred most frequently in the 21-30 years age group [11]. In another study, the fractures occurred most frequently at the age of 37 years [12]. In our study, the mean age of patients in Group I was 29.38 years and in Group II was 24.38 years.

Etiology

The etiology of facial trauma varies in different cultures and societies. The most common reason for injury is road traffic accidents [12-15]. Other causes of injury are assaults, falls, sports, and industrial accidents [12]. The most common reason for injury in our study was a road traffic accident, which is in agreement with the previous studies.

Fracture union

As mentioned previously, fracture union was assessed on clinical and radiographic criteria at the end of five weeks. Based on the clinical and radiological criteria, fracture healing was categorized as union and non-union [2]. Indistinguishable fracture lines in the radoigraph, good occlusion, immobility of fracture fragments with no discomfort during rest or activity, and no swelling were considered signs of the union. Whereas, non-union was characterized by osteolysis, fracture mobility, discomfort during rest or activity, edema/suppuration, as well as the persistence of fracture lines in the radiograph.

All eight patients in Group II and seven patients in Group I had good fracture unions. Only one patient in Group I (12.5%) had signs of non-union, including deranged occlusion, mobility at the fracture site, pain on function, and increased radiolucency in orthopantomography (at the end of five weeks). This patient had a good occlusion in the immediate post-surgical period and during the first two follow-up visits, suggesting that non-compliance with post-operative instructions was the cause for nonunion. One case in Group II had a mild occlusal discrepancy at the end of five weeks that required minimal occlusal grinding.

Wound infection and dehiscence

All patients were treated under antibiotic cover [beta-lactams (Cefotaxime/cephalexin/amoxicillin) and metronidazole]/chlorhexidine mouth rinses. None of them developed wound infection to the state of cellulitis or pus formation. Two patients (25%) in Group II developed small dehiscence (3-5 millimeters) on the third postoperative day. According to the literature, the incidence of wound dehiscence and plate exposure was always higher in the two plate groups [8,10], and our study confirms this. The causes of wound dehiscence include poor closure during surgery, smoking, trauma, and infection [10]. In our study, it was mostly related to poor oral hygiene and resolved easily with wound irrigation and resuturing (done on the fourth post-operative day) in one case. Post-operative oral hygiene instructions were re-emphasized.

Plate exposure and need for plate removal

**Figure 10:**
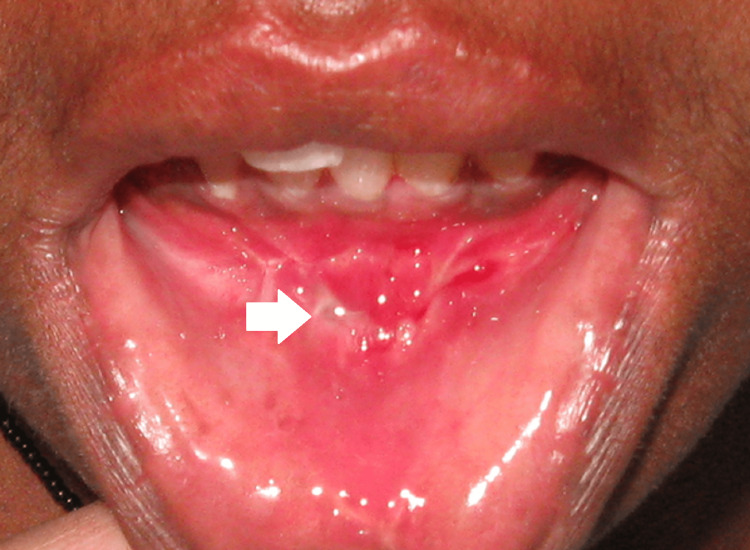
Intra-oral sinus opening seen in the lower labial vestibule

**Figure 11:**
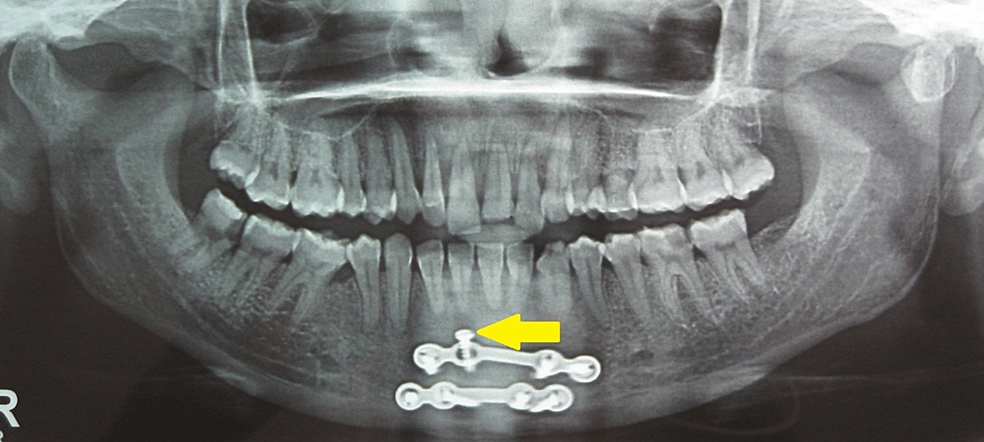
Loosening of screw seen in Group II patient

Only one patient in Group II (12.5%) developed loosening of screws and subsequent infection. Interestingly, it is one of the same patients who developed postoperative wound dehiscence. The patient presented with the complaint of discharge in relation to the previous surgical site five months postoperatively. Orthopantomograph showed radiolucency around the screws of the sup apical plate. The infected plates and screws were surgically removed under the cover of antibiotics. Since sufficient time has elapsed from primary surgery, the osseous union was not affected (Figures 10, 11).

According to the literature, the incidence of plate exposure and the need for plate removal was always higher in the two plate groups [8,10], with the subapical plate being the most commonly exposed. The findings of our study were consistent with the same. If this occurs, it is usually with the subapical plate. In our opinion, the incidence was higher in Group II due to the following reasons: the presence of a subapical plate just below the line of incision, the presence of a greater amount of foreign body, the occurrence of wound dehiscence in the immediate post-operative period predisposing them to plate infection at a later date.

Iatrogenic dental injury

**Figure 12:**
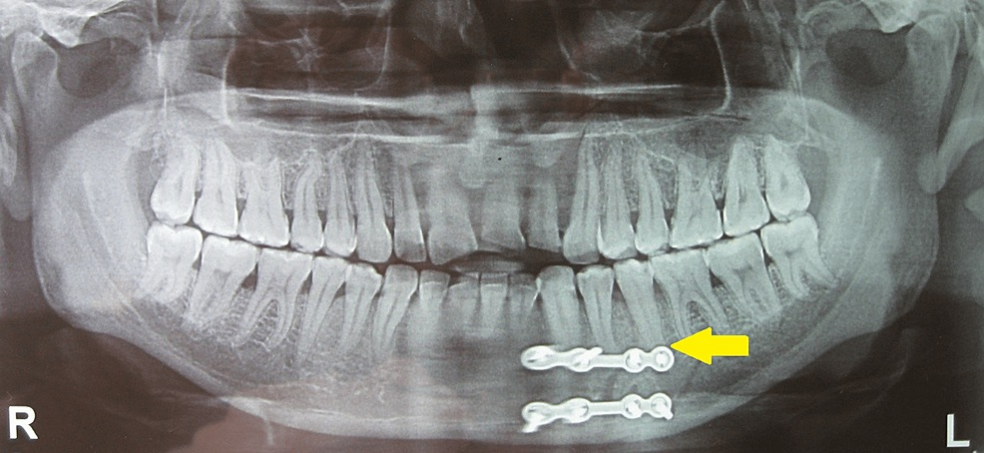
Iatrogenic dental injury in Group II patient

**Figure 13:**
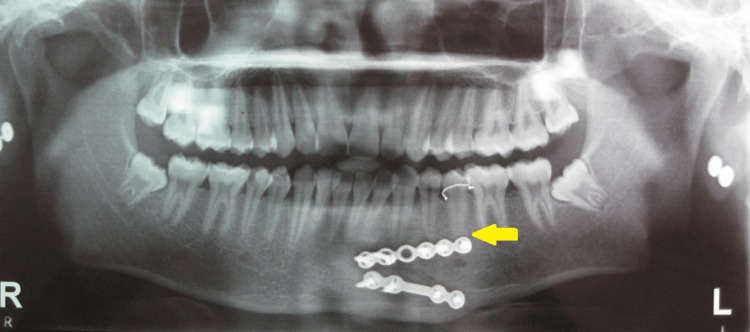
Screws of the subapical plate cause iatrogenic injury to the roots of premolar teeth

The iatrogenic dental injury was assessed only based on the postoperative radiograph; accordingly, four patients in Group II (50%) showed that the screws of their subapical plate were almost on to the roots of premolar teeth (Figures 12, 13). This was statistically higher than Group I, wherein none of the patients exhibited iatrogenic dental injury (P = 0.021). Interestingly, it occurred only when the fracture line was located in the canine-premolar region and never in the cases that were close to the symphysis. In our opinion, causes for iatrogenic dental injury are as follows: Surgeon attempting to place the plate below the root apices of the canine but above the mental foramen inadvertently landing up on to the roots of premolar, a short mandibular height that is insufficient for placing two plates, inability to judge the root apices in the premolar region as in case of anterior teeth, due to the absence of root eminence on the overlying bone.

**Figure 14:**
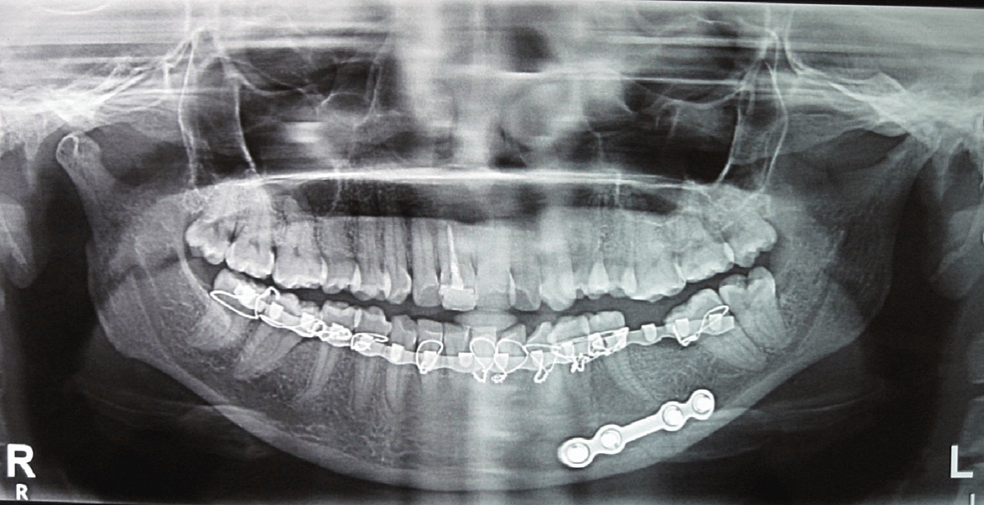
Case with minimal vertical mandibular height where the placement of single plate has avoided iatrogenic dental injury

**Figure 15:**
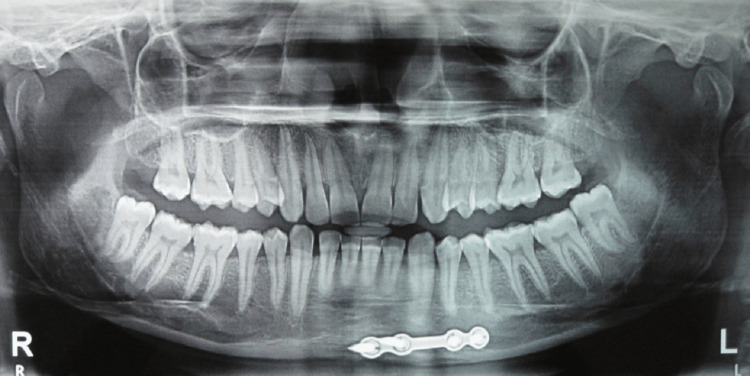
Case with minimal vertical mandibular height where the placement of single plate has avoided iatrogenic dental injury

Post-operative radiographs of patients with minimal vertical mandibular height where the placement of a single high-profile plate has avoided such an occurrence have been displayed (Figures 14, 15).

**Figure 16:**
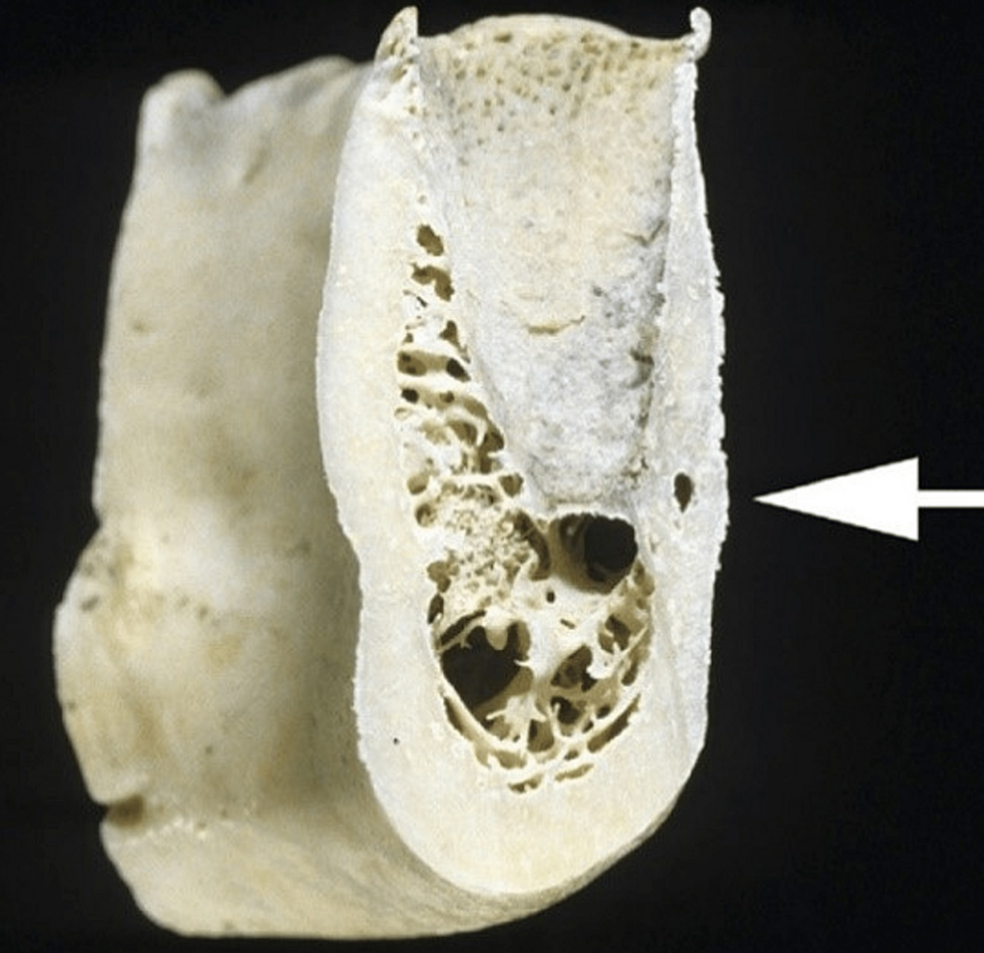
The thickness of the cortex in relation to the apex of premolar teeth

Surgeons who insert screws in a monocortical pattern appear to have the misunderstanding that these screws are always secure. Based on the examination of anatomic specimens and computed tomography images, it is found that the average buccal cortical bone thickness in the premolar region of the mandible is between 2 to 2.5 millimeters (Figure 16). The facial plate of bone is extremely thin, and the roots are immediately within. Even if there were incredibly short screws available, drilling may still harm the tooth roots underneath. Also, such short screws would provide very little bone purchase. Therefore, it would seem wise to avoid placing screws at these locations, especially in patients with minimal mandibular height, as with many female patients who are more likely to suffer iatrogenic root injuries [10].

Mental nerve paresthesia

According to the literature, the incidence of post-operative paraesthesia was always higher in the two plate groups [8], and our study confirms this. Two cases in Group I (25%) and three cases in Group II (37.5%) developed post-operative paraesthesia along the distribution of the mental nerve. These were usually transient and resolved in a period of 15 days to 2 months without any intervention. Direct mental nerve injury occurred in none of our cases, and the transient paraesthesia was all related to surgical stretching of the nerve. Placement of the plate at the lower border below the mental nerve through an intraoral approach in Group I was technically difficult and involved a greater stretching of the nerve.

Intraoperative time

It is the time consumed from the placement of the initial incision until the completion of the last suture. It usually ranged from 66 minutes to 78 minutes in Group I and from 76 minutes to 92 minutes in Group II. The average time consumption for Group I was 72 minutes, and for Group II was 84 minutes. The difference is statistically significant (P = 0.000).

## Conclusions

Biomechanics, although important, is not the only factor to be considered while selecting internal fixation schemes for fractures. In our opinion, both systems of osteosynthesis provide functionally stable fixation in terms of fracture union and occlusion; however, placing the subapical plate in the canine-premolar region, especially in patients with short mandibular height, always carries the risk of iatrogenic injury to the roots of the teeth and mental nerve. Although the screws are termed monocortical, they are much longer than the thickness of the cortex in the canine-premolar region and hence are always associated with the risk of injury to dental roots and the inferior alveolar nerve. Furthermore, the presence of a subapical plate just below the line of incision always increases the incidence of infection/dehiscence/plate exposure. The use of a single plate minimizes the surgical time, cost, and above all the amount of foreign body kept within the tissues; however, it carries a risk of fracture non-union if the patient does not comply properly with post-operative instructions.

To conclude, although both these methods of osteosynthesis provide favorable clinical outcomes, treating simple linear fractures in canine-premolar region, especially in patients with minimal mandibular height with a single miniplate and bicortical screws at the lower border of mandible supported with an arch bar at the upper border is associated with minimal complications and is always recommended. However, further studies with a larger sample size are required to ascertain the clinical benefits of one system over the other.
